# Weight changes and patterns of weight measurements in hospitalized burn patients: a contemporary analysis

**DOI:** 10.1186/s41038-018-0131-2

**Published:** 2018-10-15

**Authors:** Denisse Mendez-Romero, Audra T Clark, Alana Christie, Steven E Wolf

**Affiliations:** Department of Surgery, Division of Burn, Trauma, and Critical Care, University of Texas Southwestern Medical Center, E05514B, 5323 Harry Hines Blvd, Dallas, TX 75390 USA

**Keywords:** Burn, Weight, Nutrition, Metabolism, Critical care

## Abstract

**Background:**

Severe burn is associated with significant changes in body weight due to resuscitation volumes, fluid shifts, a hypermetabolic state, prolonged bed rest, and caloric intake. Our goal was to quantify and describe trends in weight change in patients with burns of all severities under modern treatment conditions and to identify the time points at which these changes occur.

**Methods:**

An institutional review board-approved chart review was conducted of acute burn patients treated at an American Burn Association-verified regional burn center from February 2016 to November 2016. Patients were then divided into three groups based on percent of total burn surface area (%TBSA) burn: 1–19%, 20–39%, and ≥ 40%. Weight was expressed as percent change of weight from baseline. Regression analysis was conducted on percent weight changes for each TBSA group.

**Results:**

We identified 197 burn patients with a length of stay (LOS) of ≥ 7 days. Of the study cohort, 149 had TBSA burn of 1–19%, 27 had TBSA burn of 20–39%, and 21 had TBSA burn of ≥ 40%. All groups had a majority of White male, non-Hispanic patients with mean ages between 40 and 42 years. Burn patients with > 20% TBSA burn had a median increase in weight above baseline of approximately 5 to 8% likely due to resuscitation fluids within the first week of hospitalization. Weight loss below baseline often did not exceed 10% and was more pronounced as LOS increased, mostly in patients with > 20% TBSA burn. Whereas patients with 1–19% TBSA burn on average returned to baseline weight at last measurement, patients with 20–39% TBSA and ≥ 40% TBSA burn continued a decline in weight at 4 weeks (*r*^2^ = 0.57 and 0.55, respectively) on the same trajectory.

**Conclusions:**

Burn patients with > 20% TBSA burn had an increase in weight above baseline of up to 8%, likely due to resuscitation fluids within the first week of hospitalization. Weight loss below baseline often did not exceed 10% and was more pronounced as LOS increased, mostly in patients with > 20% TBSA burn. Therefore, our patients on average, lost body weight to a lesser extent than the maximum mean loss of 22% of pre-burn weight reported prior to modern treatment conditions.

## Background

Body weight often serves as a marker of nutritional status, and large weight losses can predict mortality [1]. However, in the burn patient, many factors other than nutrition can play a role in weight changes. Severe burn is associated with significant changes in body weight due to large resuscitation volumes, fluid shifts, a hypermetabolic state, prolonged bed rest, and caloric intake.

Weight gain in the severely burned patient often follows initial fluid resuscitation, which can increase weight by up to 10–20 kg [2]. Although some variations exist among burn centers, the Parkland formula is the primary method to estimate infusion rates and lactated Ringer’s solution is the dominant resuscitation fluid [3]. Fluid resuscitation is undertaken with care since underresuscitation can lead to hypoperfusion whereas overresuscitation can cause complications such as compartment syndromes and pulmonary edema [3]. Further weight changes can be caused by fluid shifts associated with infections, ventilator support, hypoproteinemia, and elevations in aldosterone and antidiuretic hormone [4].

Weight in the burn patient is also significantly affected by the hypermetabolic response to severe burn. Although the mechanism behind hypermetabolism is not fully understood, it is defined as an increased consumption of whole-body oxygen and a resting energy expenditure over 10% above normal [5].

Hypermetabolism after burn is associated with a cascade of proinflammatory cytokines, acute-phase proteins, and catabolic hormones. These in turn raise body temperature, trigger a hyperdynamic circulation, promote hyperglycemia, and inhibit protein synthesis, correlating with an increased degree of muscle catabolism [6, 7]. It has been proposed that the breakdown of skeletal muscle might serve as a source of nitrogen to promote gluconeogenesis and wound healing in the burn patient [8]. This loss of muscle protein can last up to 1 year after burn, leading to significant loss of muscle mass, weight, and strength [9].

In the 1970s, investigators found that burn patients could lose up to 22% of their preinjury weight [10]. The magnitude of weight loss was found to be directly related to the severity of injury. Since those studies were performed, significant advances have been made in therapies to mitigate the hypermetabolic state and muscle catabolism. In the modern era of burn care, an early aggressive approach to nutrition has been adopted. Enteral nutrition (EN) is usually initiated within 24 h of injury to support caloric needs and attenuate the hypermetabolic response [11, 12]. Furthermore, the pharmacologic adjuncts propranolol and oxandrolone are currently used to blunt hypermetabolism and stimulate anabolism, respectively [13, 14]. Other methods include the early excision and closure of wounds to decrease skeletal muscle catabolism, reduce the risk of wound infection, and improve mortality [15, 16]. Regulation of body temperature, accomplished by raising the temperature in the patient’s room and utilizing wound dressings, can also reduce the hypermetabolic response.

Weight changes in the burn patient have not been well-described across time in the setting of these modern treatments. In this study, we sought to analyze weight in patients with burns of all severities across time given current therapies. Specifically, our aim was to quantify and describe trends in weight change as well as to identify the time points at which these changes occur. Furthermore, we analyzed the patterns of weight measurement throughout hospitalization. We hypothesized that the weight of burn patients would initially increase in association with resuscitation fluids and subsequently decline throughout the rest of hospitalization due to loss of muscle mass.

## Methods

An institutional review board-approved chart review was conducted of acute burn patients treated at an American Burn Association-verified regional burn center. We included all admitted burn patients from February 2016 to November 2016. Patients without a recorded weight and patients with a length of stay (LOS) less than 7 days were excluded from analysis. Demographic data including age, sex, and percent of total body surface area (%TBSA) burn were collected along with hospital LOS and all weight measurements. Patients were then divided into three groups based on %TBSA burn: 1–19%, 20–39%, and ≥ 40%. For comparison between patients, all weights were expressed as percent change of weight from baseline. Baseline was defined as weight on admission within 24 h of injury. Values are reported either as mean ± standard deviation (SD) or median [Q1–Q3]. Regression analysis was conducted on percent weight changes for each TBSA group. In regard to weight measurement, weight in our unit is automatically measured by the hospital bed with a built-in scale. A statistical software (IBM SPSS Statistics for Windows, Version 25.0) was used to perform all analyses. Significance was accepted at *p* < 0.05. This study was a retrospective review of hospital charts, and the Institutional Review Board waived the need for consent.

Patients in our unit receive an initial nutritional assessment within 24 h of admission and a secondary nutritional assessment if admitted to the intensive care unit (ICU). Nutritional follow-up of patients is at the discretion of the dietitian, with patients in more critical condition monitored more often. The decision to feed enterally is made by the dietitian based on the patient’s ability to meet appropriate caloric needs via oral feeding. In general, patients with TBSA burns of ≥ 20% and patients with an indication for operative intervention receive continuous pre-pyloric EN initiated within 24 h of admission. The prescribed daily caloric intake goal takes into account %TBSA burn and ideal body weight. Patients with TBSA burn of < 20% will usually receive 30–35 kcal/kg with 1.5–2 g/kg of protein. Patients with TBSA burn of > 20% will receive 35–40 kcal/kg with 2–2.5 g/kg of protein. In patients who are underweight, actual weight rather than ideal body weight is used in calculations of caloric needs. The standard EN formula when initiating tube feeding in adult patients is replete (1 kcal/ml; Nestle HealthCare, Bridgewater, NJ). Additionally, a glutamine supplement is ordered for all adults with > 15% TBSA burn. To monitor if a patient has received an adequate caloric intake, the dietitian compares the actual hourly rate of the tube feeding to its prescribed rate. Pharmacological adjuncts are also commonly used. Propranolol is prescribed at the discretion of the physician, and oxandrolone is started 72 h after admission in all adult patients with > 30% TBSA burn.

## Results

**Fig. 1:**
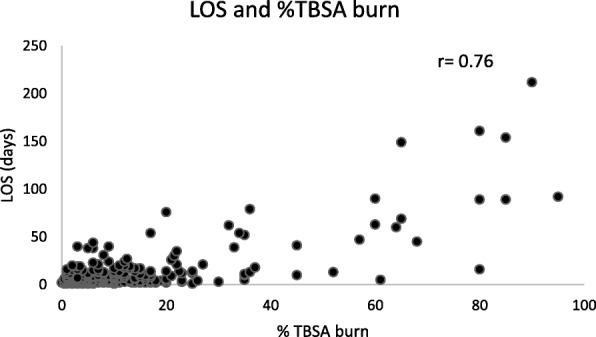
Length of stay (LOS) and percent of total body surface area (%TBSA) burn. Correlation between LOS and %TBSA burn. Pearson’s correlation coefficient (*r*) = 0.76

We identified 197 burn patients admitted between February 2016 and November 2016 who had an LOS of ≥ 7 days; 284 patients with an LOS less than 7 days were excluded. Of the study cohort, 149 had TBSA burn of 1–19%, 27 had TBSA burn of 20–39%, and 21 had TBSA burn of ≥ 40%. Demographics of the study population are shown in Table 1. In general, baseline characteristics were consistent among TBSA groups. All groups had a majority of White male, non-Hispanic patients with mean ages between 40 and 42 years. Median %TBSA burn was 6% [Q1–Q3: 2–10], 27% [Q1–Q3: 22–35] and 65% [Q1–Q3: 60–80] in the groups of TBSA 1–19%, 20–39%, and ≥ 40%, respectively. LOS increased as burn severity worsened with a median LOS of 9 days [Q1–Q3: 7–14], 18 days [Q1–Q3: 12–37], and 63 days [Q1–Q3: 34–90] for the three groups. Figure 1 shows the significant positive correlation between %TBSA and LOS (Pearson’s correlation coefficient (*r*) = 0.76). Furthermore, patients who remained in the hospital longer were weighed more often throughout their hospitalization. On average, patients with an LOS of 7–14 days, 15–30 days, 31–60 days, and > 60 days had their weight measured on 11.3, 19.4, 33.7, and 46.6% of their total inpatient days, respectively (Table 2).

**Table 1:** Demographics of study population by percent of total body surface area (%TBSA) burn group

	LOS ≥ 7 days (*n* = 197)	%TBSA 1–19% (*n* = 149)	%TBSA 20–39% (*n* = 27)	%TBSA ≥ 40% (*n* = 21)
Gender (%)				
Female	27	30	19	19
Male	73	70	81	81
Age, years (mean ± SD)	41 ± 20	42 ± 22	40 ± 18	41 ± 18
Race (%)				
White	66	62	78	72
Black	28	32	15	14
Asian	0	1	0	0
Unknown	6	5	7	14
Ethnicity (%)				
Hispanic	21	21	19	29
Non-Hispanic	72	73	78	57
Unknown	7	6	3	14
%TBSA (median, [IQR])	8 [4–17]	6 [2–10]	27 [22–35]	65 [60–80]
LOS, days (median, [IQR])	11 [8–18.8]	9 [7–14]	18 [12–37]	63 [34–90]

*LOS* length of stay, *IQR* interquartile range, *SD* standard deviation

**Table 2:** Records of the frequency of weight measurement during hospitalization

	LOS (days)			
	7-14	15-30	31-60	>60
Patients (n)	130	35	16	16
Percent of inpatient days in which weight was recorded	11.3	19.4	33.7	46.6

*LOS* length of stay

**Fig. 2:**
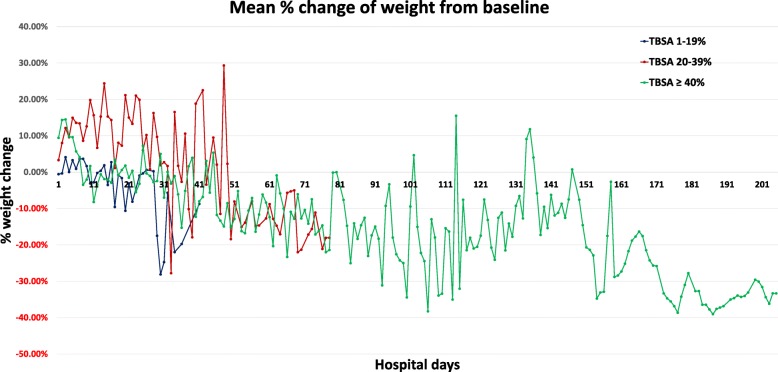
Mean percent change of weight from baseline. Mean percent change of weight from baseline throughout hospitalization in total body surface area (TBSA) groups 1–19%, 20–39%, and ≥ 40%. Baseline was defined as the weight recorded on hospital day 1. There was a significant difference in median percent weight change at last measurement between TBSA groups (*p* < 0.001)

**Fig. 3:**
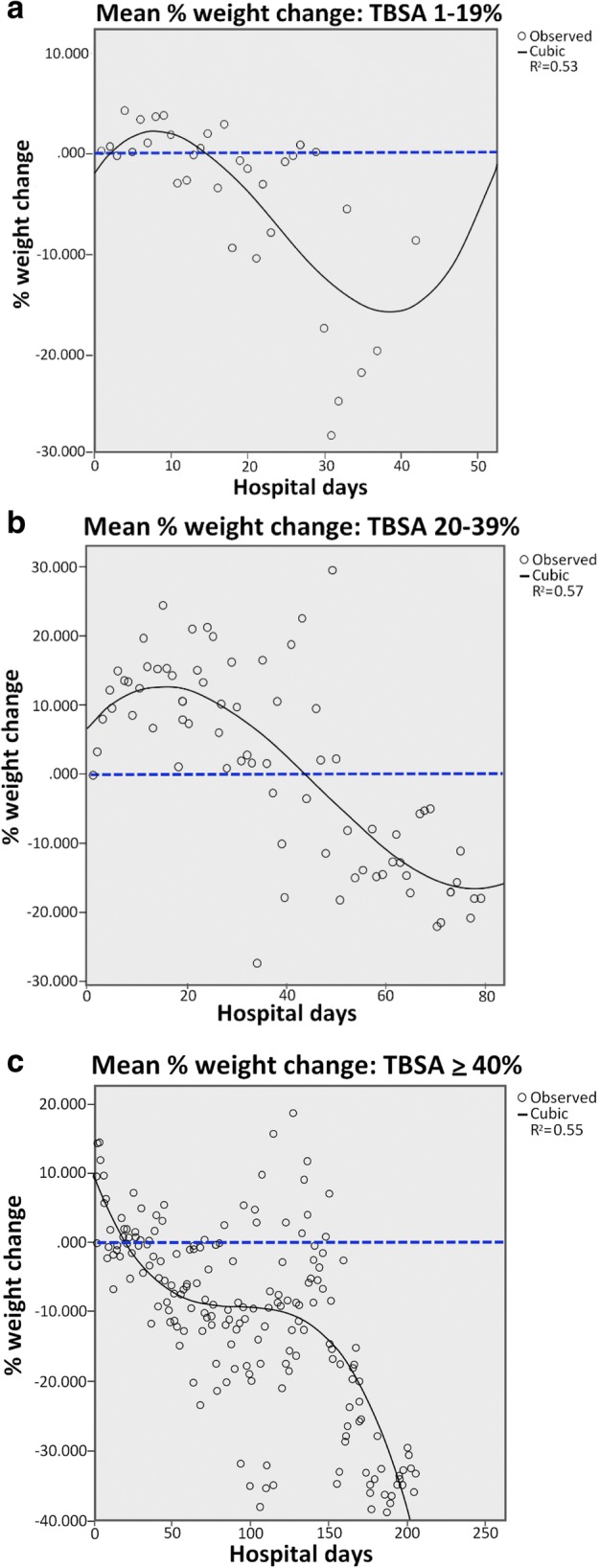
Mean percent weight change in total body surface area (TBSA) groups 1–19%, 20–39%, and ≥ 40%. Regression analysis of weight trends in the groups of TBSA 1–19% (**a**), 20–39% (**b**), and ≥ 40% (**c**). The weights best followed third-order regression with *r*^2^ values of 0.53, 0.57, and 0.55, respectively

Figure 2 depicts mean weight change from baseline in each TBSA group throughout hospitalization. In general, we found a loss of weight below baseline in all three groups that became more pronounced as LOS increased. The trends in weight change of each group followed third-order regression with *r*^2^ values of 0.53, 0.57, and 0.55 for TBSA groups of 1–19%, 20–39%, and ≥ 40%, respectively (Fig. 3a–c).

Percent changes of weight during select time points throughout hospitalization are shown in Table 3. Patients with a TBSA burn of 1–19% showed essentially no weight change from baseline at hospital day 7 and 15. The weight in this group then showed a decrease below baseline of − 11.6% [− 14.5 to − 8.6] at day 30. Only one patient remained hospitalized at day 45, with a median weight loss below baseline of − 8.7%. Patients with a TBSA burn of 20–39% showed an increase in weight above baseline of 8.3% [2.1–16.8] and 5.6% [3.6–7.4] at hospital day 7 and 15 followed by a decrease in weight below baseline of − 2.9% [− 8.5 to − 1.4] at day 30. Two patients remained hospitalized at day 45 and 60, with median weight losses of − 8.6% and − 8.5% below baseline. Patients with a TBSA burn of 40% or greater demonstrated a median increase in weight of 5.3% [− 2.9–11.5] and 0.2% [− 7.4–10] above baseline at hospital days 7 and 15 followed by a decrease in weight below baseline of − 1.4% [− 6.5–4], − 7.6% [− 15–6.8], − 7.2% [− 10.9 to − 2.9], − 4.2% [− 18.2–1.7], and − 7.8% [− 16.3 to − 0.3] at hospital day 30, 45, 60, 90, and 120, respectively. The maximum LOS in this group was 213 days, involving a patient who showed a maximum weight loss below baseline of − 39% at day 188.

**Table 3:** Median change of weight (median, [IQR]) from baseline by percent of total body surface area (%TBSA) burn group during selected time points throughout hospitalization

Groups	LOS (days)							
	7	15	30	45	60	90	105	120
TBSA 1-19%	0.0 [- 0.3-0.9]	0.0 [- 2.5-0.3]	-11.6 [- 14.5 to - 8.6]	-8.7	-	-	-	-
TBSA 20-39%	8.3 [2.1-16.8]	5.6 [3.6-7.4]	-2.9 [- 8.5 to - 1.4]	- 8.6 [- 13 to - 4.2]	- 8.5 [- 10.6 to -6.4]	-	-	-
TBSA ≥40%	5.3 [- 2.9-11.5]	0.2 [- 7.4-10]	- 1.4 [- 6.5-4]	-7.6 [- 15-6.8]	- 7.2 [- 10.9 to -2.9]	- 4.2 [- 18.2-1.7]	3.8 [- 5.9-4.6]	- 7.8 [- 16.3 to - 0.3]

*LOS* length of stay, *IQR* interquartile range

We found a significant difference in median percent weight change at last measurement between TBSA groups as determined by Kruskal–Wallis H Test (*p* < 0.001). Last measurement differed between TBSA groups due to different LOS (TBSA 1–19%: 5.4 ± 6.8 days; TBSA 20–39%: 16 ± 20.3 days; TBSA 40–100%: 75.6 ± 63.8 days). Patients with a TBSA burn of 1–19% essentially returned to baseline weight with a median weight change from baseline of 0.0% [− 0.5–0.7]. Patients with a TBSA burn of 20–39% had mostly gained weight at last measurement with a median increase of 5.1% [− 1.9–14.2]. Of note, although, as shown in Fig. 2, weight trended downward in this group as LOS increased, the weight in these patients was only followed for a median of 8 days. Since more than 90% of these patients had an LOS greater than 8 days, the recorded last measurement does not reflect weight measurements near the end of a hospital stay but rather an earlier measurement. Finally, patients with a TBSA burn of ≥ 40% had primarily lost weight at last measurement with a median decrease of − 3.8% [− 14.9–4.4] below baseline.

## Discussion

In this study, the weight of burn patients in all three TBSA groups trended downwards after an initial increase in weight above baseline during the first days of hospitalization. Weight loss became more pronounced as LOS increased, which was primarily in patients with more severe burns with a TBSA of 40% or greater. Patients with TBSA 20–39% also showed more pronounced weight loss as LOS increased; however, this group was only followed for a median of 8 days. Therefore, it is difficult to predict the trend in weight change at subsequent time points in this group. The initial weight increase was likely the result of resuscitation fluids. As expected, patients with more severe burns (TBSA 20–39% and ≥40%) presumably received larger volumes of fluid leading to greater weight increases of approximately 5–10% within the first week of hospitalization compared to patients with burns covering a TBSA of less than 20%. Weight decrease below baseline was potentially caused by loss of skeletal muscle. Studies have shown that catecholamines associated with the hypermetabolic response in burn injury are elevated in proportion to TBSA burn [15, 16]. Jeschke et al. demonstrated that children with > 40% TBSA burn significantly lost more body weight, lean body mass, and muscle protein than those with < 40% TBSA burn [17]. Similarly, we observed larger weight loss in patients with larger %TBSA burn with a failure to return to baseline levels. However, in our study, patients with < 40% TBSA burn also showed smaller weight losses below baseline. Whether a hypermetabolic response to burn injury is also at work in this weight decrease is unclear. It is possible that in patients with smaller burns, prolonged bed rest could play a larger role in the loss of skeletal muscle.

Although the causes of weight loss in the burn patient have not been completely elucidated, it is likely due to multiple factors. Hypermetabolism induced by burn injury leads to muscle catabolism and subsequent loss of lean body mass. This hypermetabolic state can be ameliorated through nutritional, surgical (e.g., early excision and burn closure), and pharmacological therapies. Decreased muscular activity due to prolonged bed rest and immobilization also causes muscle loss. Bed rest is thought to result in muscle wasting through decreased muscle protein synthesis rather than increased protein breakdown [18]. However, even minimal activity and increasing the muscle force production while the patient is supine may reduce the loss of lean body mass [19]. Suboptimal nutritional support has also been implicated in the weight loss of burn patients. Sudenis et al. found that burn patients in a large adult burn center received fewer calories than the calories prescribed by a dietitian on every day of hospitalization studied [20]. The percentage of prescribed calories that patients actually received ranged from 19 to 91% as LOS increased [20]. The major causes for the discrepancies between prescribed and received calories included the interruption of feedings for surgery and gradual increases in the feed rate before reaching the prescribed goal [20]. Prescribing more calories than the patient needs may be one way to combat this caloric deficit. However, this strategy may lead to overfeeding, which has been shown to increase the accumulation of fat mass, especially in the liver, rather than lean mass [7, 21].

Previous reports on body weight in burn patients, mostly from the 1970s, described weight losses of up to 22% of pre-burn levels at 8 weeks in patients with TBSA burn of ≥ 40%, likely caused by hypermetabolism, which was worse in patients with more severe burns [10]. These studies were conducted at a time where nutritional therapies significantly differed from current practices. Early nutritional support of the burn patient relied on total parenteral nutrition (TPN) using feeding formulas developed from little data [22]. Later studies found that the intestinal mucosal atrophy induced by TPN was associated with an elevated acute-phase response, mobilization of peripheral amino acids, and increased production of proinflammatory mediators [23]. Today, TPN has been mostly replaced by EN since EN directly stimulates the intestinal mucosa, maintains gut-associated immune function, and delivers nutrients directly to the liver, reducing hyperosmolarity and hyperglycemia. Furthermore, providing EN within 24 h of burn injury has been shown to mitigate the hypermetabolic response, promote muscle mass maintenance, decrease intensive care unit stay, improve wound healing, and decrease the risk of wound infection [11, 12]. In the setting of these modern therapies, we found that our patients on average still lose body weight but to a lesser extent than the maximum mean loss of 22% of pre-burn weight reported in the 1970s. Most weight losses in our study population did not exceed 10%. Nonetheless, weight loss of this magnitude may still be detrimental to the patient, since a weight loss of approximately 10% has been found to impair immunity and a weight loss greater than 10% may result in decreased healing and possibly death [24].

This study has shown that weight loss in the burn patient is still present despite current nutritional and rehabilitation therapies. Further investigation is warranted regarding the molecular mechanisms of skeletal muscle loss and therapies to combat the loss of lean body mass. In the severely burned patient, loss of lean body mass may be greater than what is apparent from weight changes alone. Zdolsek et al. found that total body water may remain increased weeks after burn injury, masking a loss of muscle [25]. On the other hand, increased feeding may not prevent the erosion of lean body mass and instead lead to increased accumulation of body fat [7]. Additionally, how the dynamics of weight loss and loss of skeletal muscle differ in the setting of obesity remains to be explored. After severe trauma, obese patients may present with an increased secretion of insulin, magnified proteolysis, significant resistance to lipolysis, and a greater degree of inflammation [26]. For these reasons, continuous monitoring of long-term weight trends and nutritional status are necessary. However, since large weight loss was only seen in patients with > 20% TBSA burn, there is a potential for risk-stratification and intensive weight monitoring only in these patients.

Of note, this study is limited by the manner of weight measurement. Since weight was measured automatically by hospital beds with built-in scales, variance was potentially introduced by patients changing beds and by the amount of dressings present. We have no data as to how often patients changed bed, and wound dressings were not weighed separately. Further variability may be present since there is no specific protocol that determines when body weight is measured. These factors may account for the fluctuations in weight change seen among our different TBSA burn groups.

## Conclusions

In summary, we found that burn patients with > 20% TBSA burn had a median increase in weight above baseline of up to 8%, likely due to resuscitation fluids within the first week of hospitalization. Weight loss below baseline often did not exceed 10% and was more pronounced as LOS increased, mostly in patients with > 20% TBSA burn. Therefore, our patients, on average, lost body weight to a lesser extent than the maximum mean loss of 22% of pre-burn weight reported in the 1970s. Whereas patients with 1–19% TBSA burn on average returned to baseline weight at last measurement, patients with ≥ 40% TBSA burn did not. These findings indicate the need for careful monitoring of weight trends in burn patients and a potential for new therapies to prevent weight loss and its associated morbidities.

## Abbreviations

EN: Enteral nutrition
IQR: Interquartile range
LOS: Length of stay
SD: Standard deviation
TBSA: Total body surface area
TPN: Total parenteral nutrition
